# Supplementary material for: Mechanisms of azole antifungal resistance in clinical isolates of Candida tropicalis
Source: PLoS One. 2022 Jul 12;17(7):e0269721. doi: 10.1371/journal.pone.0269721 (PMC9275685; doi:10.1371/journal.pone.0269721)
Supplement: S1 File — (DOCX) [file pone.0269721.s001.docx]

**Mechanisms of azole antifungal resistance in clinical isolates of *Candida tropicalis***

Saikat Paul, Dipika Shaw, Himanshu Joshi, Shreya Singh, Arunaloke Chakrabarti, Shivaprakash M. Rudramurthy, Anup K Ghosh*

Department of Medical Microbiology, Postgraduate Institute of Medical Education and Research (PGIMER), Chandigarh - 160012, India.

*** Corresponding author**.

**Correspondence**: **Dr. Anup K Ghosh**
 Additional Professor

Department of Medical Microbiology,

Postgraduate Institute of Medical Education and Research (PGIMER),

Chandigarh 160012, India.
 Email: anupkg3@gmail.com

Tel.: +91 172 2755156.

Fax: +91 172 2744401.

**S1 Table. Sequences of overlapping primers for mutation analysis of target genes**

| **Primer name** | **Sequence (5'->3')** | **Starts** | **Stops** | **Product length** |
| --- | --- | --- | --- | --- |
| *ERG1*-F1 | AACACAACTTTCAACAAGTCCC | -122 | -101 | 927 |
| *ERG1*-R1 | TGTGGAAGCAAAAGCACACA | 804 | 785 |  |
| *ERG1*-F2 | CGAAGTTGTTGAATACCATGCCA | 561 | 583 | 1070 |
| *ERG1*-R2 | TGAGGCGACATTGTGTCAAG | 1630 | 1611 |  |
| *ERG3*-F1 | ATTCCATTCACTATTGCCTGGTC | -200 | -178 | 836 |
| *ERG3*-R1 | AGTGCCTTATAACCACCAGTAGA | 635 | 613 |  |
| *ERG3*-F2 | TGTCTTTGGAAATCGGTTTGGC | 497 | 518 | 898 |
| *ERG3*-R2 | TCAAACGCCTGATTAAACTAACC | 1394 | 1372 |  |
| *ERG11*-F1 | TCACAGTTATAGACCCACAAGG | -71 | -50 | 878 |
| *ERG11*-R1 | TCACCGCTTTCTCTTCTTCTCT | 806 | 785 |  |
| *ERG11*-F2 | AAGGTTTCACCCCAATCAACTT | 677 | 698 | 1113 |
| *ERG11*-R2 | CGACTGAAACGTATACCGCGA | 1789 | 1769 |  |
| *TAC1*-F1 | GGGAAGTCGTGCAGATTTGA | -229 | -210 | 963 |
| *TAC1*-R1 | CAAGTGGTAAGTTTCTTATGGGTGT | 733 | 709 |  |
| *TAC1*-F2 | TCTCAAGGGATTCTTTAGCATGGA | 647 | 670 | 985 |
| *TAC1*-R2 | TGGATCTGTCGCAATATGCCT | 1631 | 1611 |  |
| *TAC1*-F3 | AGTCAGGTAAACCGCCAATGA | 1430 | 1450 | 992 |
| *TAC1*-R3 | AGGAAACTTTGTCTCGGCTTCT | 2421 | 2400 |  |
| *TAC1*-F4 | TCATTAGCAGAATCGCAACGAG | 2155 | 2176 | 833 |
| *TAC1*-R4 | CACAAACATTGAGCCTTGCGT | 2987 | 2967 |  |
| *UPC2P*-F1 | TCCCTAAATCGTTCCTTTCGT | -201 | -181 | 998 |
| *UPC2P*-R1 | CAGATACTGCCAGTGATTTTCTTG | 796 | 773 |  |
| *UPC2P*-F2 | TTGGCAACGTTGGCTCAATTAC | 619 | 640 | 940 |
| *UPC2P*-R2 | TAGCAACTAATGCGTCCGTGT | 1558 | 1538 |  |
| *UPC2P*-F3 | GCCTTTAGTGCTACTCACTTGTC | 1420 | 1442 | 1110 |
| *UPC2P*-R3 | CCACACTTATCTCTTTATGCATGTG | 2529 | 2505 |  |

F1, F2, F3, and F4 = Forward primer for fragment 1, 2, 3, and 4; R1, R2, R3, and R4 = Reverse primer for fragment 1, 2, 3, and 4

**S2 Table. Sequences of primers for the expression analysis of target genes**

| **Gene symbol** | **Gene Name** | **Accession number** | **Sequence (5'->3') forward and reverse** | **Amplicon length (bp)** |
| --- | --- | --- | --- | --- |
| *CDR1* | Candida drug resistance gene | XM_002548263.1 | TCGCCGTTTGCTGAAGAAGA  GCAATCCCCAATTTCGATGGT | 140 |
| *CDR2* | Candida drug resistance gene | XM_002547994.1 | AAGGTGCAATCCAAAAGGGTG  CTCAATATCACTGGGTGCTCCA | 101 |
| *CDR3* | Candida drug resistance gene | XM_002545459.1 | CAAAAATCACAAGGGCAGCCA  TGCTGCAGTTTGGGTATCGT | 135 |
| *MDR1* | Multi drug resistance gene | XM_002548069.1 | GCAGTTACCTCATCTGGAGCA  GCACCAAACAATGGGAACACA | 149 |
| *ERG1* | Squalene epoxidase | [XM_002551139.1](https://www.ncbi.nlm.nih.gov/nucleotide/XM_002551139.1?report=genbank&log$=nucltop&blast_rank=1&RID=TYW6C11X014) | GAAAGAGTTCGTGGTGTTGCT  AACGGTTGCTTCAACAGCAG | 102 |
| *ERG2* | Δ^8,7^-isomerase | XM_002550035.1 | CGGATGGAAATGCCACTGCT  ATAGTACCCATGGCACCACC | 130 |
| *ERG3* | Δ^5,6^-desaturase | [XM_002550136.1](https://www.ncbi.nlm.nih.gov/nucleotide/XM_002550136.1?report=genbank&log$=nucltop&blast_rank=1&RID=TYVWY1EC01R) | TTGGCAACTAGAGCCATTCCA  AGTGCCTTATAACCACCAGTAGA | 122 |
| *ERG11* | Lanosterol C14 alpha-demethylase | XM_002550939.1 | TTGCCATTCGGTGGTGGTAG  ACATCTGGAACCTTATCACCGTT | 128 |
| *ERG24* | C14-reductase | XM_002547516.1 | ACGACTGGTTCATTGGTAGAGA  TGATGATGAGCACAACTAAGATTGA | 124 |
| *HMG* | HMG-CoA reductase | XM_002550004.1 | CAAGGCATCGACTTCATCGC  CATTCCCATAAGCCGCACAG | 134 |
| *MRR1* | Multidrug resistance regulator | XM_002547926.1 | TCACACCACCTTGTCCAACTC  GGAGATGGGGTCCCTCTAGTAT | 112 |
| *TAC1* | Transcriptional activator of *C*DR genes | XM_002550963.1 | AGTCAGGTAAACCGCCAATG  ACCGAAGTCAATGCCGAAGAT | 121 |
| *UPC2* | Transcription factor of *ERG11* | XM_002548816.1 | GCTAATGGCCCACAACCAAA  GGCGGTAGCTTCTGGACTTG | 123 |
| *HSP90* | Heat shock protein 90 | XM_002548439.1 | CCAGCAGAGGAGGAAGAACA  TGGTAGTGGTAGATGGTTGGT | 121 |
| *HOG1* | Mitogen-activated protein kinase involved in osmoregulation | XM_002546830.1 | GAATTAGTGCTGCCGAAGCC  CCACTGGCAAATCTGCATCA | 120 |
| *MKC1* | Mitogen-activated protein kinase | XM_002546243.1 | CGCTGGGTTCATGACAGAGT  TTCCTCCCAAAAGCTCTGCT | 134 |
| *SOD1* | Superoxide dismutase | XM_002545332.1 | GTCATCAGTTGGACCACCGT  TGCCTTGAGAGGTTTCCACA | 112 |
| *EF1* | Elongation factor 1α | [XM_002547480.1](https://www.ncbi.nlm.nih.gov/nucleotide/XM_002547480.1?report=genbank&log$=nucltop&blast_rank=1&RID=TYWCWH7N01R) | GGTCAAACCAGAGAACACGC  TTCTTCAAATCTGTTTTTGTCCCA | 111 |

**S3 Table. Model quality score wild type and mutant lanosterol C14 alpha demethylase (*ERG11p*)**

| **Protein** | **Ramachandran plot (In %)** | | | | **Overall**  **𝐺-factor**** | **Verify 3D** | **ERRAT** |
| --- | --- | --- | --- | --- | --- | --- | --- |
|  | **Most favoured**  **regions** | **Additional**  **allowed**  **regions** | **Generously**  **allowed**  **regions** | **Disallowed**  **Regions** |  |  |  |
| **Wild type** | 90.2 | 8.5 | 0.9 | 0.4 | -0.10 | 87.50% | 94.37 |
| **Mutant** | 89.8 | 9.2 | 0.9 | 0.2 | -0.16 | 87.50% | 93.7751 |

****Overall G factor:** acceptable values of G-factor in PROCHECK is between 0 and -0.5 with the best model displaying values close to zero.

**S4 Table. *ERG1* and *UPC2* gene sequence analysis among resistant and susceptible isolates**

|  | **Mutation in *ERG1* gene** | **Amino acid alteration** | **Mutation in *UPC2* gene** | | **Amino acid alteration** | |
| --- | --- | --- | --- | --- | --- | --- |
|  | **220** | **74** | **503** | **751** | **168** | **251** |
| **Reference sequence (MYA-3404)** | A | N | T | G | L | A |
| **Resistant isolates without mutation** | A | N | T | G | L | A |
| **Resistant isolates with mutation** | C | H | C | A | P | T |
| **Susceptible isolates** | A | N | T | G | L | A |

N: Asparagine; H: Histidine; L: Leucine; P: Proline; A: Alanine; T: Threonine


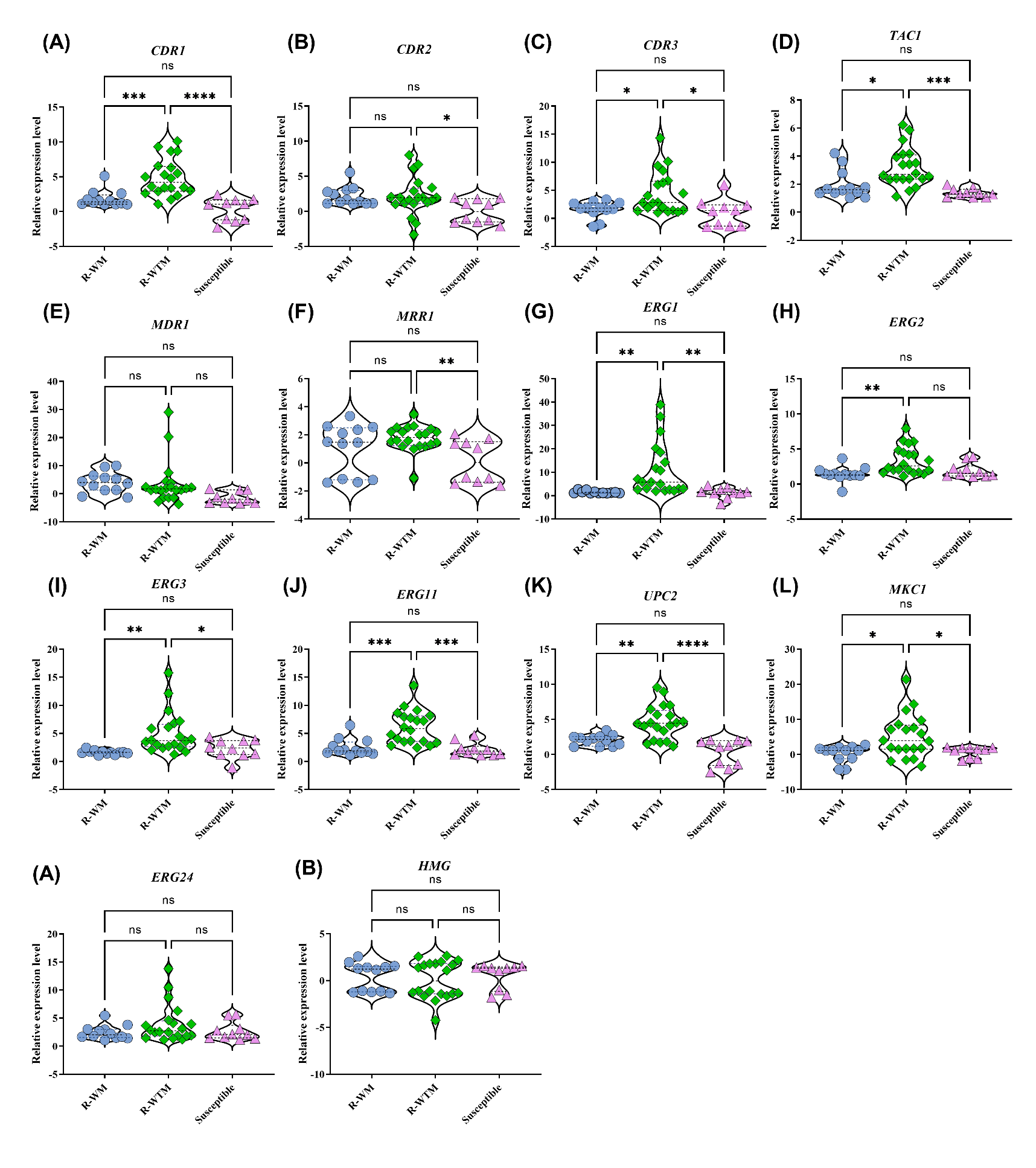


**S1 Fig. Scatter dot plots presenting the inducible expression of *HMG* gene among R-WM and R-WTM and S isolates.**


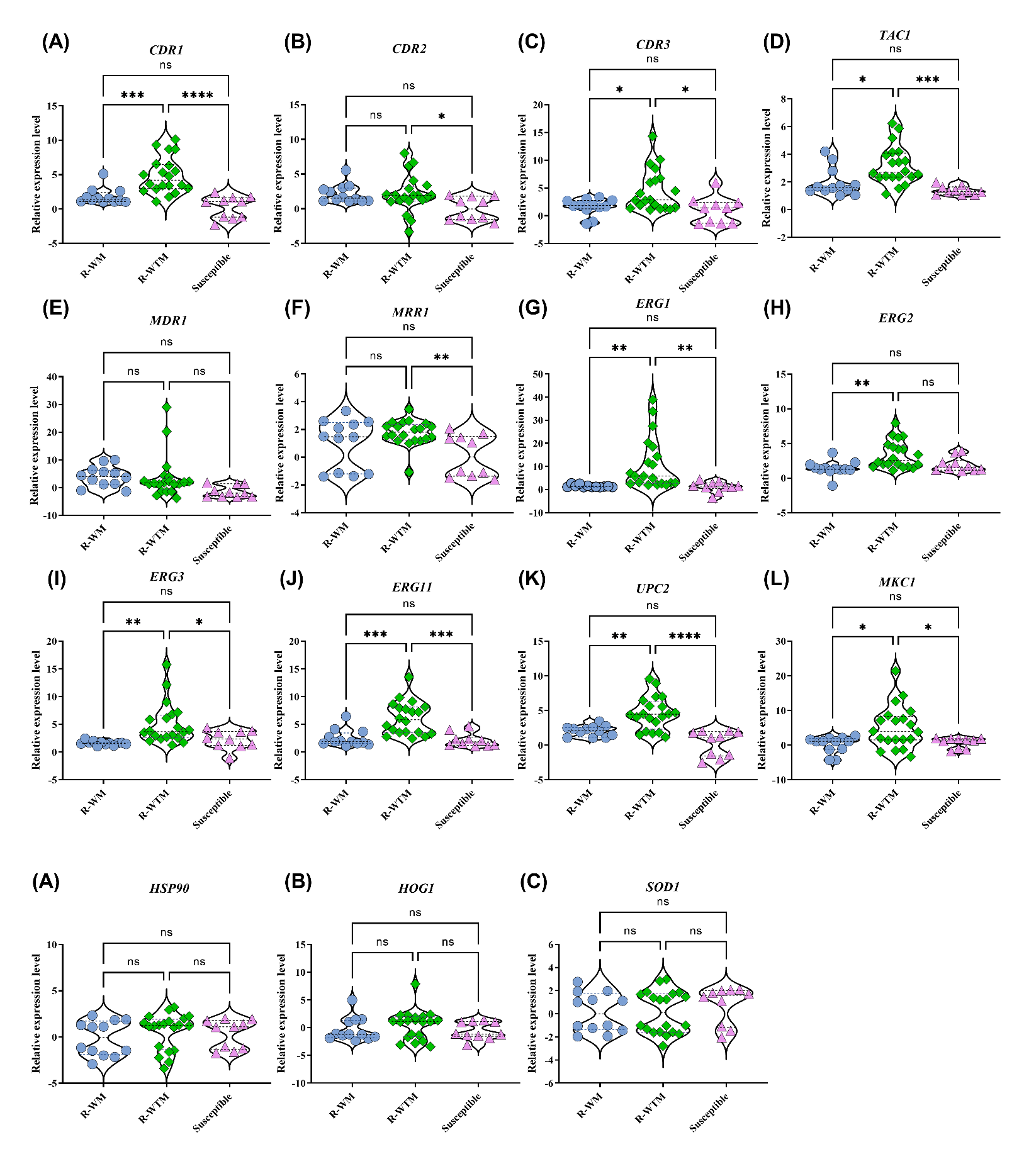


**S2 Fig. Scatter dot plots depicting the inducible expression of stress responsive pathway genes (*HSP90, HOG1,* and *SOD1*) among R-WM and R-WTM and S isolates. * p<0.05 and NS=Non-Significant.**


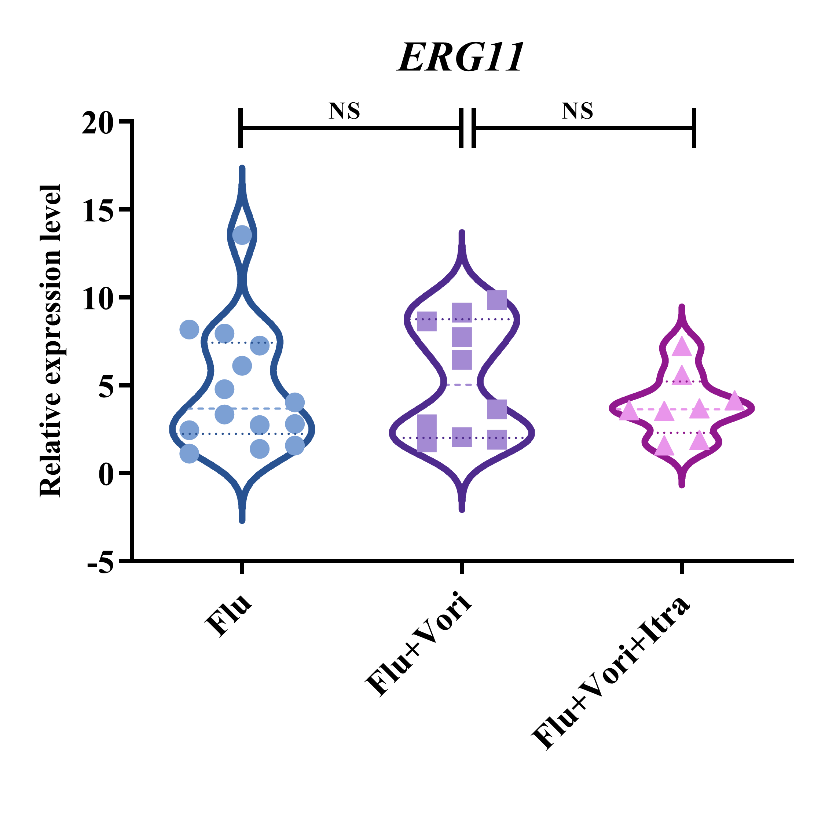


**S3 Fig. Plots representing the inducible expression of *ERG11* gene among R-WM and R-WTM and S isolates. * p<0.05 and NS=Non Significant.**

**S1 Material and Methods. Homology modelling and model Refinement**

The ERG11 protein sequence for *Candia tropicalis* was retrieved by using ExPASy Translate tool. To identify a template for construction of tertiary structure the sequence was matched against PDB (Protein Data Bank) database [1] using BLASTp program [2]. PDB entry with maximum score and coverage was selected as template for Homology Modelling using Modeller 9.25 [3]. Mutagenesis was achieved using PyMOL [4] and the most favored confirmation was selected. The wild and mutant structures were further subjected to energy minimization using GROMOS96 in Swiss-PdbViewer [5] and further refinement were achieved using UCSF Chimera1.15 [6].

**S2 Material and Methods. Model quality assessment and validation**

The refined model was validated for its quality, internal consistency and reliability by a number of computational tools. PROCEHCK [7] was used to check the stereo- chemical quality of the model, which quantifies the residues in the available zones of Ramachandran plot. A Ramachandran plot provides the position of the torsion angles phi (φ) and psi (ψ) between Cα-C and N-Cα atoms of the residues contained in a peptide. ERRAT tool [8], which finds the overall quality factor of the protein, was used to check the statistics of non-bonded interactions between different atom types. Verify3D program [9] determines the compatibility of the atomic model (3D) with its own amino acid sequence (1D) where a high Verify3D profile score indicates the high quality of a protein model. All the above analyses were carried out using structural analysis and verification server (SAVES) (http://nihserver.mbi.ucla.edu/SAVES/). After each model and loop refinement step, the above model quality assessment programs were employed to check the error at each residue in the protein. This process was repeated iteratively until the most geometrically and energetically stable structural conformation was attained.

Effect of mutation was analyzed using ERIS web server [10] was used to infer the effect of mutations on the structural stability of protein by calculating the ΔΔG value (Gibbs free energy). Substitution of amino acid will be considered destabilizing if the ΔΔG is >0 and vice versa.

**S3 Material and Methods. Molecular docking study**

Prior to the docking calculation the heme cofactor from the template 5V5Z from Chain A, Lanosterol 14-alpha demethylase from Candida albicans was extracted and merged with the native and the mutated protein structures. The models were energy minimized and stable conformation of the native as well as mutated protein structure was used as receptor molecule for the docking study to probe the binding free energy between the drug molecules (Fluconazole and Voriconazole) and receptor using AutoDock 4.2 [11]. Autodock Tools (ADT) [11] was used for optimizing the receptor and ligand molecules. Polar hydrogens, Kollman charges and AD4 type of atoms were added for preparation of the receptor molecule, while Gasteiger charges were added to the drug molecules with maximum numbers of active torsions. AutoGrid4 was used to prepare a grid map of interaction energies around the heme cofactor located in the active site. Throughout the docking simulation the receptor molecule was set to rigid and rests of the docking parameters were set to default values using Lamarckian Genetic Algorithm (LGA). Thirty different poses were generated for each drug molecules and scored using AutoDock 4.2 scoring functions [12] and were ranked according to their docked energy. AutoDock Tools and PyMOL were used for post docking analysis.

**S4 Material and Methods. References**

1. Berman HM. The Protein Data Bank. Nucleic Acids Res. 2000;28: 235–242. doi:10.1093/nar/28.1.235

2. Altschul SF, Gish W, Miller W, Myers EW, Lipman DJ. Basic local alignment search tool. J Mol Biol. 1990;215: 403–410. doi:10.1016/S0022-2836(05)80360-2

3. Šali A, Blundell TL. Comparative Protein Modelling by Satisfaction of Spatial Restraints. J Mol Biol. 1993;234: 779–815. doi:10.1006/jmbi.1993.1626

4. DeLano WL. The PyMOL Molecular Graphics System, Version 2.3. Schrödinger LLC. 2020.

5. Guex N, Peitsch MC. SWISS-MODEL and the Swiss-Pdb Viewer: An environment for comparative protein modeling. Electrophoresis. 1997;18: 2714–2723. doi:10.1002/elps.1150181505

6. Pettersen EF, Goddard TD, Huang CC, Couch GS, Greenblatt DM, Meng EC, et al. UCSF Chimera?A visualization system for exploratory research and analysis. J Comput Chem. 2004;25: 1605–1612. doi:10.1002/jcc.20084

7. Laskowski RA, MacArthur MW, Moss DS, Thornton JM. PROCHECK: a program to check the stereochemical quality of protein structures. J Appl Crystallogr. 1993;26: 283–291. doi:10.1107/S0021889892009944

8. Colovos C, Yeates TO. Verification of protein structures: Patterns of nonbonded atomic interactions. Protein Sci. 1993;2: 1511–1519. doi:10.1002/pro.5560020916

9. Lüthy R, Bowie JU, Eisenberg D. Assessment of protein models with three-dimensional profiles. Nature. 1992;356: 83–85. doi:10.1038/356083a0

10. Yin S, Ding F, Dokholyan N V. Eris: an automated estimator of protein stability. Nat Methods. 2007;4: 466–467. doi:10.1038/nmeth0607-466

11. Morris GM, Huey R, Lindstrom W, Sanner MF, Belew RK, Goodsell DS, et al. AutoDock4 and AutoDockTools4: Automated docking with selective receptor flexibility. J Comput Chem. 2009;30: 2785–91. doi:10.1002/jcc.21256

12. Madeswaran A, Umamaheswari M, Asokkumar K, Sivashanmugam T, Subhadradevi V, Jagannath P. In Silico docking studies of lipoxygenase inhibitory activity of commercially available flavonoids. J Comput Method Mol Des. 2011;1: 65–72. Available: http://scholarsresearchlibrary.com/archive.html
